# Mucosal immunization with a low-energy electron inactivated respiratory syncytial virus vaccine protects mice without Th2 immune bias

**DOI:** 10.3389/fimmu.2024.1382318

**Published:** 2024-04-05

**Authors:** Valentina Eberlein, Sophia Rosencrantz, Julia Finkensieper, Joana Kira Besecke, Yaser Mansuroglu, Jan-Christopher Kamp, Franziska Lange, Jennifer Dressman, Simone Schopf, Christina Hesse, Martin Thoma, Jasmin Fertey, Sebastian Ulbert, Thomas Grunwald

**Affiliations:** ^1^ Fraunhofer Institute for Cell Therapy and Immunology IZI, Leipzig, Germany; ^2^ Fraunhofer Cluster of Excellence Immune-Mediated Diseases (CIMD), Frankfurt am Main, Germany; ^3^ Fraunhofer Institute for Applied Polymer Research (IAP), Potsdam, Germany; ^4^ Fraunhofer Institute for Organic Electronics, Electron Beam and Plasma Technology (FEP), Dresden, Germany; ^5^ Fraunhofer Institute for Translational Medicine and Pharmacology (ITMP), Frankfurt, Germany; ^6^ Department of Respiratory Medicine and Infectious Diseases, Hannover Medical School, Hannover, Germany; ^7^ Member of the German Center for Lung Research (DZL), Biomedical Research in Endstage and Obstructive Lung Disease Hannover (BREATH), Hannover, Germany; ^8^ Fraunhofer Institute for Toxicology and Experimental Medicine (ITEM), Hannover, Germany; ^9^ Fraunhofer Institute for Manufacturing Engineering and Automation (IPA), Stuttgart, Germany

**Keywords:** Respiratory Syncytial Virus (RSV), mucosal vaccination, formulation, low-energy electron irradiation (LEEI), mucosal immunity

## Abstract

The respiratory syncytial virus (RSV) is a leading cause of acute lower respiratory tract infections associated with numerous hospitalizations. Recently, intramuscular (i.m.) vaccines against RSV have been approved for elderly and pregnant women. Noninvasive mucosal vaccination, e.g., by inhalation, offers an alternative against respiratory pathogens like RSV. Effective mucosal vaccines induce local immune responses, potentially resulting in the efficient and fast elimination of respiratory viruses after natural infection. To investigate this immune response to an RSV challenge, low-energy electron inactivated RSV (LEEI-RSV) was formulated with phosphatidylcholine-liposomes (PC-LEEI-RSV) or 1,2-dioleoyl-3-trimethylammonium-propane and 1,2-dioleoyl-sn-glycero-3-phosphoethanolamine (DD-LEEI-RSV) for vaccination of mice intranasally. As controls, LEEI-RSV and formalin-inactivated-RSV (FI-RSV) were used *via* i.m. vaccination. The RSV-specific immunogenicity of the different vaccines and their protective efficacy were analyzed. RSV-specific IgA antibodies and a statistically significant reduction in viral load upon challenge were detected in mucosal DD-LEEI-RSV-vaccinated animals. Alhydrogel-adjuvanted LEEI-RSV i.m. showed a Th2-bias with enhanced IgE, eosinophils, and lung histopathology comparable to FI-RSV. These effects were absent when applying the mucosal vaccines highlighting the potential of DD-LEEI-RSV as an RSV vaccine candidate and the improved performance of this mucosal vaccine candidate.

## Graphical Abstract

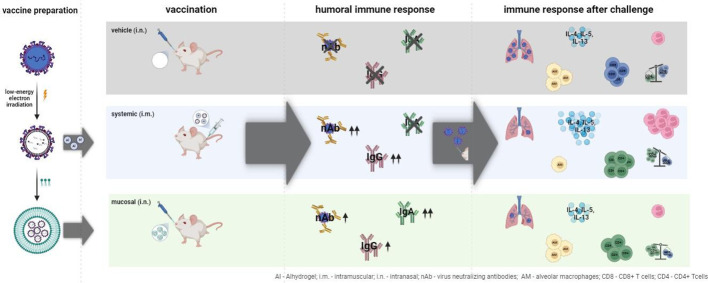

## 1 Introduction

A major threat for children and elderly are infections with the human respiratory syncytial virus (RSV), leading to numerous hospitalizations and deaths (1–4). RSV is highly infectious and seasonal and leads to upper and lower respiratory tract infections (URTI and LRTI, respectively), whereby in 2019 approximately 33 million LRTI and 101,400 deaths in children under 6 years were RSV-associated (1, 2). Just recently, two vaccines against RSV for adults that are based on recombinant RSV-F protein and given intramuscularly were approved by the FDA (5, 6).

RSV is a respiratory virus infecting the airway mucosa, and therefore an immune response directly at the site of infection may be beneficial (7, 8). The mucosal immune response includes antibodies of the IgA subtype as well as tissue resident immune cells that directly attack pathogens (7, 8). When using systemic vaccination, such as intramuscular (i.m.) injection, usually only weak mucosa-specific responses are induced (7). Mucosal immunizations have been reported to induce both strong mucosal and systemic immune responses (8–10). The mucosal application routes—e.g., nasal, oral, or vaginal—may not only lead to more efficient and suitable protection but also enhance vaccination acceptance in the population, as they are atraumatic (7, 11). For the development of a mucosal vaccine, it should be considered that the mucosa presents a barrier to prevent the intrusion of pathogens (7). Therefore, high antigen doses, mucosa-specific formulation—to enable immune recognition, or repeated applications are necessary to prevent vaccine interception by the mucosa (7, 11). Replication competent systems, such as live attenuated vaccines (LAAVs) or viral vectors, have shown striking protective immune responses after mucosal application (8, 9, 12–15). However, despite their promising protective effects, they hold substantial risks such as reversion to a virulent pathogen, reactogenicity, or vector immunity (16–18). These biological risks could be circumvented with the use of an inactivated, non-replicating vaccine.

We have previously shown that a low-energy electrone irradiation (LEEI) RSV (LEEI-RSV) in a suitable formulation induced protective immune responses against RSV after mucosal application (19). Packaging with a phosphatidylcholine (PC) formulation of LEEI-RSV was crucial for protective efficacy, reducing the viral load in the lung of challenged mice by 170-fold (19). LEEI was used as a non-toxic and non-probe-harming inactivation method with improved safety compared to chemical inactivation or other radiation methods. It has been used successfully for the generation of several viral, bacterial, and parasitic vaccine candidates (19–25).

In this study, we tested different formulations and analyzed the immune response by detecting secreted cytokines and immune cell compositions in the bronchoalveolar lavage (BAL) of mucosally vaccinated mice after RSV challenge and compared it with mice vaccinated i.m. with formalin-inactivated (FI-) RSV or LEEI-RSV. Here we found an expected Th2 bias in the Alhydrogel-adjuvanted i.m. groups, but IgA induction solely after mucosal vaccination. These results will lead to a better understanding of the mechanism of immune responses triggered by mucosal vaccination, especially with respect to IgA antibody induction, blood and BAL cell composition, and cytokine secretion.

## 2 Materials and methods

### 2.1 Cell culture and virus production

Type 2 human epithelial cells (HEp-2; ATCC, USA) were used for RSV-A production and assays and Vero E6 cells (ATCC) for RSV-B production. The cells were maintained in Dulbecco’s modified Eagle’s medium (DMEM) with GlutaMAX (Thermo Fisher Scientific, Germany), containing 10% heat-inactivated fetal calf serum and antibiotics (100 IU/mL penicillin and 100 µg/mL streptomycin; Thermo Fisher Scientific) at 37°C with 5% CO_2_.

RSV laboratory strain A-long (VR-26™) or strain B (VR-1400™) was obtained from ATCC. M. Peeples and P. Collins (NIH, USA) kindly provided the recombinant RSV-A expressing GFP (rgRSV). Virus propagation and titer determination were performed as described (12, 26, 27).

### 2.2 Virus inactivation

The RSV-A samples were irradiated in a research LEEI prototype (21) in a module based on a microfluidic system as previously described (25). In short, RSV [in PBS with 12% (w/v) trehalose] was filled into a disposable syringe which was inserted into the system and connected to a microfluidic chip (MFC) made of titan with eight parallel channels (180 µm in depth) milled into it. The MFC was sealed with a 25-µm titan foil welded onto it. For inactivation, LEEI with 300 keV and either 1 mA or 1.25 mA was applied at a transportation velocity of 40 mm/s and cooled to ~10°C during the process. The irradiated sample was collected in a new syringe. The controls underwent the same process without LEEI.

For chemical inactivation, the virus was prepared in a way comparable to “Lot#100” (28). In short, the same purified RSV stock as used for LEEI-inactivation was diluted in DMEM and incubated with 0.025% (v/v) formaldehyde (Thermo Fisher Scientific) for 96 h at 37°C and with 5% CO_2_. The virus was pelleted by ultracentrifugation (50,000 × *g*, 1 h, 4°C) in a SureSpin 630 swing-out rotor (Thermo Fisher Scientific). After resuspension in MEM (Thermo Fisher Scientific), the virus was precipitated with 4 mg/mL Alhdydrogel (InvivoGen, USA) for 30 min, followed with centrifugation at 1,000 × *g* for 30 min. The FI-RSV was resuspended in MEM and stored at -80°C.

Inactivation of all material was confirmed as described (20, 21). For better comparison, a single RSV batch was used for all LEEI-RSV-immunized groups in the animal experiment.

### 2.3 RSV surface antigen conservation after inactivation

To examine the conservation of RSV antigenicity, dot blot analysis and enzyme-linked immunosorbent assays (ELISAs) were performed. ELISAs were performed as described with the same antibodies as dot blots (19–21). For dot blot, 2 µL of each inactivated RSV sample, with respective untreated controls, were put on a nitrocellulose membrane (GE Healthcare Life Sciences, Germany). The membranes were blocked with 5% (w/v) skimmed milk powder (Carl Roth, Germany) in PBS-T (PBS with 0.05% Tween 20) (Bio&Sell, Germany; Carl Roth) for 1 h and incubated with a monoclonal antibody (mAb) recognizing RSV-F [18F12, 1:400 (26)], a mAb recognizing the prefusion form of RSV-F (RSV-preF) (D25, 1:1,000; Cambridge Biologics, USA), or a mAb recognizing RSV-G (8C5, 1:500; Invitrogen, USA) in 2% (w/v) skimmed milk in PBS-T at 4°C overnight. After washing three times with PBS-T, the membranes were incubated with the respective secondary antibody: for 18F12 and 8C5, the peroxidase AffiniPure™ sheep anti-mouse IgG (H+L)-horseradish peroxidase (HRP) antibody (Dianova, Germany) diluted 1:500, and for D25, the goat anti-human IgG HRP-conjugated 1:20,000 (Dianova) for 1 h. The membranes were washed, developed with enhanced chemiluminescent substrate (Pierce, USA), and imaged on a CELVIN^®^ chemiluminescent imager (Biostep, Germany).

### 2.4 Liposome production and virus packaging

Phosphatidylcholine (PC, LIPOID GmbH, Germany) liposome formulations were prepared as previously described (19). LEEI-RSV was mixed in a ratio of 1:5 (PC : LEEI-RSV) with the liposome formulation and vortexed for 15 s. This material was kept at 4°C.

Cationic liposomes were prepared as described with some modifications (29, 30). In a small round-bottom flask, equal amounts of DOTAP (1,2-dioleoyl-3-trimethylammonium-propane) and DOPE (1,2-dioleoyl-sn-glycero-3-phosphoethanolamine; both lipids from Avanti Polar Lipids *via* Sigma-Aldrich, Germany) (DD) were mixed in chloroform and dried in a rotary evaporator followed by 1 h at high vacuum to yield a homogenous lipid film. The film was hydrated in sterile HEPES buffer (20 mM HEPES–NaOH, pH 7.4) and vortexed for 15–20 min. The mixture was extruded 10 times using a mini extruder (Avanti Polar Lipids) equipped with a 100-nm membrane. A rather high total lipid concentration of 14 mg/mL was chosen [29] to prevent strong virus dilution during formulation. The liposomes were analyzed by dynamic light scattering (DLS) using Zetasizer (Malvern, Germany) and Prometheus Panta (Nanotemper, Germany). DD liposomes have mean particle sizes (dh) of 96 and 85 nm, depending on the DLS device, with a polydispersity index below 0.2, proving good mono-dispersity (**Supplementary Figure S1**). The extrusion step yielded small, homogenous, and unilamellar liposomes ensuring reproduceable formulation. LEEI-RSV and liposomes were mixed directly before usage in a 1:5 ratio (DD : LEEI-RSV) and incubated for 1.5 h on ice, inverting it from time to time.

### 2.5 Human precision-cut lung slices

The use of lung tissue was approved by the Ethics Committee of the Hannover Medical School (MHH, Hannover, Germany) and was in compliance with “The Code of Ethics of the World Medical Association” (renewed on 2015/04/22, number 2701-2015). All patients gave written informed consent for the use of their lung tissue for research. Human precision-cut lung slices (PCLS) were prepared as described previously (31). PCLS containing airways were treated with different concentrations of DD-LEEI-RSV and cultured in DMEM/F12 with penicillin and streptomycin (10,000 U/mL, Gibco) at 37°C with 5% CO_2_ for up to 72 h.

Tissue viability was assessed using the lactate dehydrogenase (LDH)-based Cytotoxicity Detection Kit (Roche, Switzerland) and the metabolic activity-based Cell Proliferation Reagent WST-1 (Roche). Adverse immunomodulatory effects were assessed by IL-6 and TNF-α in the supernatants by using ELISA (RnD, DuoSet, USA). All kits and reagents were applied at the manufacturer’s recommendations.

### 2.6 Immunization of mice and humoral immune response

**Figure 1:**
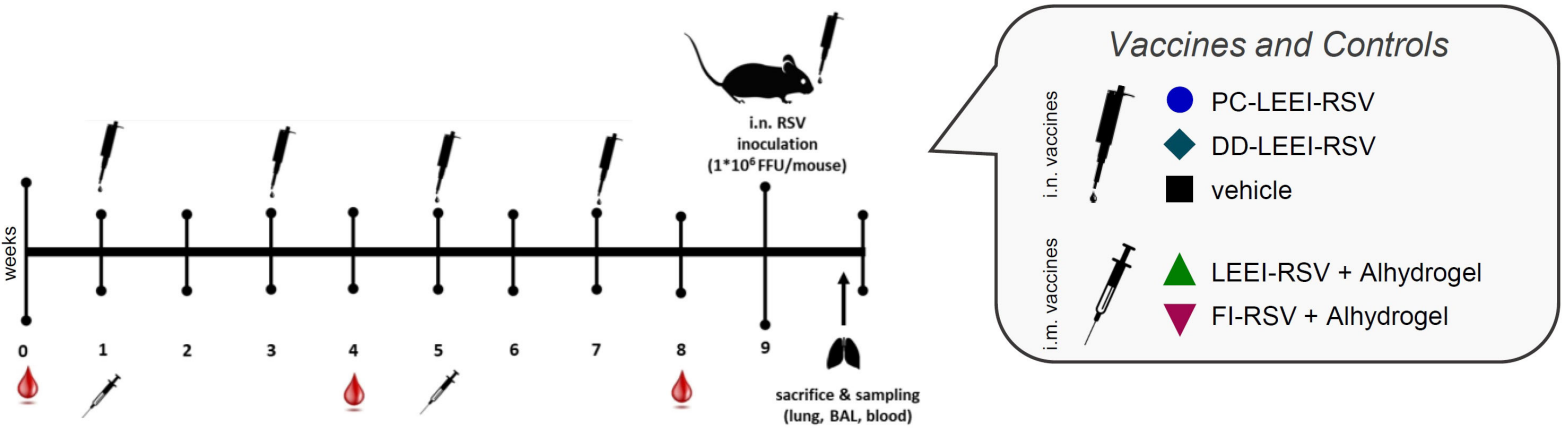
Scheme of the vaccination schedule and infection with RSV. BALB/c mice were vaccinated using homologous vaccine regiments. For the formulated LEEI-RSV vaccine with different liposomes for the formulated LEEI-RSV vaccines with different liposomes the application route was intranasal (i.n.), as well as for the vehicle group which recieved PBS with 12% trehalose. As presented, the intranasal application was performed four times in total, i.e., every 2 weeks. For comparison, the vaccinees received LEEI-RSV (LEEI-RSV i.m.) or formalin-inactivated RSV (FI-RSV i.m.) two times intramuscularly (i.m.) in a 4-week interval, both with the Alhydrogel adjuvant. Blood samples were collected for immunogenicity analysis at the indicated timepoints. At 8 weeks after the first vaccination, the animals were challenged with 10^6^ FFU (focus-forming units) RSV and scored for 5 days to test the protective efficacy. At 5 days after challenge, the mice were euthanized and final blood samples were taken; bronchoalveolar lavage (BAL) was performed as well, whereby the left lung lobes was separated after inflation for histological analysis. The remaining lung tissue was homogenized for further analysis.

Female BALB/c mice (Charles River Laboratories, Germany) were maintained under a specific pathogen-free environment in isolated ventilated cages. The animal experiments were carried out according to EU Directive 2010/63/EU and approved by local authorities. Groups of six mice, 8 weeks of age, were vaccinated as shown in **Figure 1**. Homologous i.m. vaccination with Alhdydrogel-adjuvanted LEEI-RSV (LEEI-RSV i.m.) or FI-RSV (FI-RSV i.m.) was done by injecting 50 µL in each hind leg under isoflurane anesthesia (total volume per vaccination: 100 µL). The LEEI-RSV i.m. was a control for protection, and the FI-RSV i.m. was a control for adverse effects. Intranasal (i.n.) homologous vaccines were applied with PC-formulated LEEI-RSV (PC-LEEI-RSV), DD-formulated LEEI-RSV (DD-LEEI-RSV), or the vehicle [PBS with 12% (w/v) trehalose] in a volume of 50 µL under light isoflurane anesthesia. For all vaccines, the RSV titer was ~10^8^ FFU/mL. Blood for the serum samples was collected 1 week before and 3 weeks after i.m. prime and i.m. boost vaccination for antibody analysis.

Serum RSV-neutralizing antibody titers were determined as described (19).

The analysis of RSV-A-binding antibodies was modified based on our previously published protocol (19). Serum dilution was 1:1,000 for IgG and 1:250 for IgA. For IgA detection, the goat anti-mouse IgA cross-adsorbed HRP secondary antibody (Invitrogen) was used in a 1:300 dilution. To analyze RSV-B-binding antibodies, the same procedure was used with 10^5^ FFU RSV-B/well coated.

### 2.7 RSV challenge and sample preparation

The mice were challenged 4 weeks after i.m. and 2 weeks after last i.n. boost-immunization, respectively, with 10^6^ FFU RSV-A-long at 50 µL per animal under light anesthesia (**Figure 1**). At 5 days after infection, the mice were bled and euthanized, and the lungs were flushed with PBS to access the BAL, whereby at the first lung inflation the left lobes were separated for histological analysis. Pulmonary histopathology was performed as described (20) and evaluated in a blinded manner by the external veterinarian pathologist Prof. Dr. Robert Klopfleisch (Free University, Berlin, Germany). The BALs were centrifuged for 5 min at 500 × g and 4°C, separating the BAL cells from the BAL fluid (BALF) (27). The BAL cells were used for flow cytometric analysis and the BALF for cytokine and antibody analysis. Binding antibodies were analyzed as described in Section 2.6 with a 1:100 BALF dilution. The right lung lobes were used for the analysis of RSV copies *via* RT-qPCR as described (19, 27).

### 2.8 Flow cytometry

The BAL cells were stained with the antibody mix (**Table 1**) in FACS buffer (PBS, 0.5% BSA, 2 mM EDTA) for 10 min at 4°C, washed with 2 mL FACS buffer, and centrifuged at 500 × *g* for 5 min at 4°C. The blood samples were diluted 1:2 with PBS and incubated with the antibody mix (**Table 1**). After 10 min of incubation at 4°C, 1 mL of 1× BD FACS™ Lysing Solution (BD Biosciences, USA) was added and incubated for 10 min at room temperature in the dark for erythrocyte lysis. Then, 2 mL PBS was added and centrifuged at 350 × *g* for 5 min; washing was repeated with 3.5 mL PBS twice. The washed BAL and blood cell pellets were resuspended in an appropriate volume of FACS buffer and measured on BD™ Canto II with the DIVA software (BD Biosciences). Analysis was performed with the FlowJo™ Software (BD Biosciences). The gating strategy for blood cells is presented in **Supplementary Figure S2** and for BAL cells in **Supplementary Figure S3**.

**Table 1:** Antibodies for staining-mastermix; all antibodies were diluted at 1:50 and acquired from Miltenyi Biotec (Germany).

Antigen	Fluorophore	Clone
CD45	VioGreen	REA737
CD11b	VioBlue	REA592
CD11c	APC-Vio770	REA754
MHCII	PerCP	REA813
CD3	APC-Vio770	REA641
Ly6G	APC-Vio770	REA526
CD49b	APC-Vio770	REA981
CD4	PE-Vio770	REA641
CD8	VioBrightFITC	REA601
CD170	PE	REA798

The cytokine levels in the BALF of infected mice were determined using BD™ CBA Enhanced Sensitivity Flex Sets according to the manufacturer’s instructions and analyzed with the corresponding BDTM software. The BALF was diluted 1:3 in assay diluent.

### 2.9 Statistical analysis

Statistical analysis was performed using GraphPad Prism Version 6.07. The tests used and significance values are indicated in the figure legends and tables.

## 3 Results

### 3.1 Improved surface conservation of LEEI-RSV compared to FI-RSV

To generate high-titer vaccine preparations, RSV was inactivated by LEEI using a MFC, which enables the processing of small batches requiring less virus material (25). After infections on HEp2 cells, the material treated with 1.25 mA and showed no CPE in three passages was considered inactivated and used for further testing, whereas the 1 mA-treated material was not completely inactivated and therefore discarded (**Supplementary Figure S4A**). FI-RSV was used as a control for chemical inactivation after proving complete inactivation (**Supplementary Figure S4A**).

**Figure 2:**
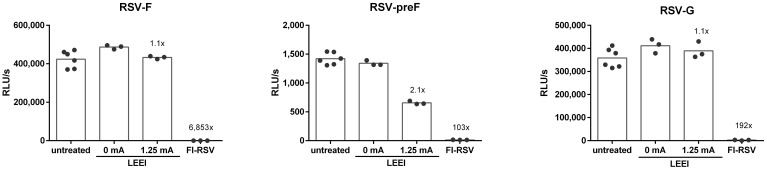
Conservation of RSV surface proteins after irradiation. The conservation of RSV-F (18F12), RSV-preF (D25), and RSV-G (8C5) after inactivation was measured in untreated control (untreated), the LEEI process control (0 mA), and after inactivation with 1.25 mA LEEI or formalin (FI) by ELISA. Shown are LEEI-RSV inactivated in MFC and formalin-inactivated RSV (FI-RSV) used in the animal-experiments in comparison to the respective controls. The bars indicate the mean of each group; every dot is a replicate, and shown is a representative experiment out of two. The calculated fold reduction to the respective 0 mA control is indicated above each group.

The conservation of the RSV surface proteins RSV-F and RSV-G was analyzed by using ELISA. In addition, the conservation of the prefusion RSV-F (RSV-preF) was assessed since the presence of RSV-preF is crucial for the induction of balanced immune responses after vaccination (32). Whereby the surface antigens in the process control were comparable to the untreated RSV, irradiation with 1.25 mA caused a 2.1-fold reduction for RSV-preF (**Figure 2**), while for FI-RSV the surface protein antigenicity was highly reduced (6,853-fold RSV-F, 103-fold RSV-preF, and 192-fold RSV-G) (**Figure 2**). The dot blot analysis showed comparable results whereby no signals for FI-RSV were detectable (**Supplementary Figure S4B**).

### 3.2 Protection in mice

To test the efficacy of our mucosal vaccine candidates, we used the commonly accepted RSV infection BALB/c mouse model (33). The mice were vaccinated either i.n. four times bi-weekly, including the vehicle control, or, for comparison, twice i.m. using a homologous prime-boost regimen with a 4-week interval (**Figure 1**).

#### 3.2.1 IgA antibodies only in mucosally vaccinated mice

**Figure 3:**
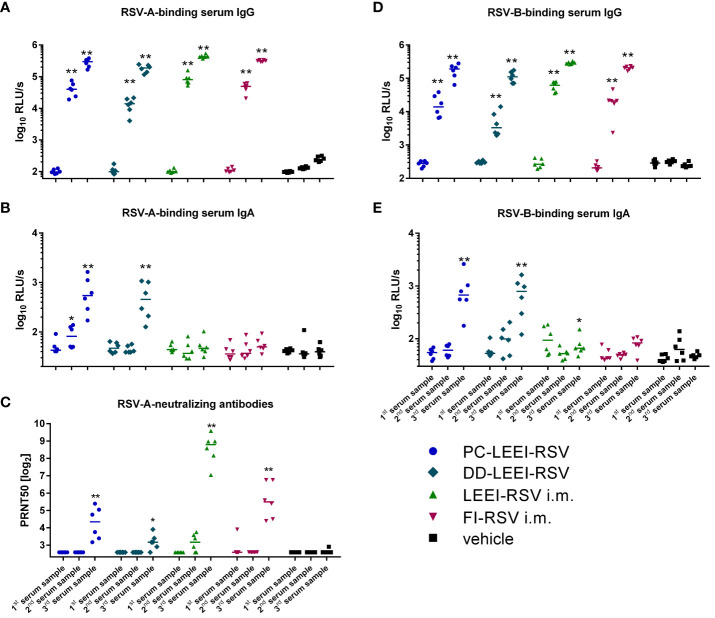
Systemic humoral immune response of immunized animals. BALB/c mice were vaccinated as described in **Figure 1**. The humoral immune responses before and after vaccination were detected by RSV-binding and RSV-neutralizing antibodies in serum samples. Before (first serum sample) and at 3 weeks after the i.m. prime (second serum sample) and boost (third serum sample) vaccination or, respectively, 1 week after two i.n. (second serum sample) and after four i.n. vaccinations (third serum sample), blood samples were collected to monitor the systemic humoral immune responses. RSV-binding serum IgG antibodies **(A, D)** and IgA antibodies **(B, E)** were tested by using ELISA either against an RSV-A **(A, B)** or an RSV-B **(D, E)** subtype. Furthermore, 50% plaque neutralization titers (PRNT50) in sera were tested in a microneutralization assay against RSV-A **(C)**. Every dot is the mean of the duplicate of one animal, and shown is a representative experiment **(A, B, D, E)**. In **(C)**, every dot represents the mean of two to three separate measurements in duplicates of one animal. Statistical evaluation was performed by using Mann–Whitney test in comparison to the respective vehicle-vaccinated animal (indicated above each group). (*p ≤ 0.05, **p ≤ 0.01; LOD, limit of detection for the virus neutralization titer at 1:6; *n* = 6; relative light units per second = RLU/s).

Systemic IgG antibodies against the RSV-A strain were quantified. Since RSV-A was used for vaccine preparation, we expected anamnestic immune responses against the identical antigens after all vaccinations. A statistically significant induction of IgG-antibodies compared to the vehicle control group could be observed in all vaccination groups in the second and third serum samples (**Figure 3A**; **Supplementary Table S1**).

IgA antibodies are the main immunoglobulins in mucosal immune responses, and RSV-A-binding IgAs could only be detected in the serum of the two groups with i.n. vaccination (**Figure 3B**), but not in the i.m. vaccinated groups. The IgA induction in the i.n. groups was statistically significant compared to the vehicle (17-fold PC-LEEI-RSV and 13-fold DD-LEEI-RSV) and to i.m. vaccinated groups (13-fold PC-LEEI-RSV and 10-fold DD-LEEI-RSV) (**Figure 3B**; **Supplementary Table S1**).

Compared to the vehicle-immunized group, all groups showed significantly higher amounts of systemic neutralizing antibodies against RSV-A after boost vaccination (**Figure 3C**). LEEI-RSV i.m. induced the highest neutralizing antibody titers in the sera that were significantly higher than all other groups by a factor between 7- and 69-fold (**Supplementary Table S2**).

To analyze the broad antibody response and cross-reactivity after vaccination, the antibody binding capacity to RSV-B subtype was additionally tested. The RSV-B-binding serum-IgG antibodies of all vaccinated groups showed a statistically significant increase in the second and third serum samples compared to the vehicle group (**Figure 3D**). The RSV-B-binding IgA antibodies were significantly induced after mucosal vaccination with DD-LEEI-RSV (17-fold) and PC-LEEI-RSV (20-fold) compared to the vehicle group (**Figure 3E**). The LEEI-RSV i.m. third serum samples also had significantly higher amounts compared to the vehicle group, but this 1.6-fold induction can be considered negligible. Both mucosally vaccinated groups had statistically significant 12-fold (PC-LEEI-RSV) and 10-fold (DD-LEEI-RSV) higher IgA amounts compared to the LEEI-RSV i.m. group (**Supplementary Table S2**).

#### 3.2.2 Viral load reduction in DD-LEEI-RSV and LEEI-RSV i.m. groups

**Figure 4:**
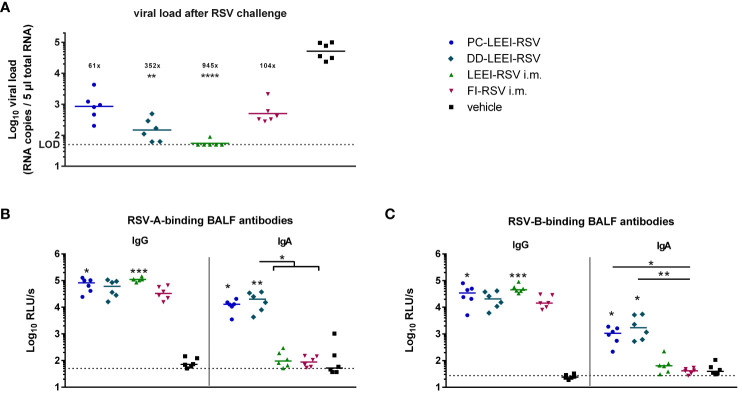
Viral load in lungs and RSV-binding antibodies in the BALF after RSV challenge. BALB/c mice were treated as described in **Figure 1**. At 4 weeks after i.m. boost immunization, the animals were challenged with 10^6^ FFU RSV-A per mouse. At 5 days after challenge, the mice were euthanized, and lung tissue and bronchoalveolar lavage fluid (BALF) were collected. The RSV load was measured in the lungs *via* qRT-PCR **(A)**, and RSV-A- **(B)** or RSV-B- **(C)** binding antibodies in BALF were quantified by using ELISA. Shown is the viral copy number of each animal measured in duplicate with the corresponding geometric mean of each group **(A)**. The calculated viral load reduction to the untreated control is indicated above each group. In **(B, C)**, every dot is the mean of the duplicate of one animal, and also shown is a representative experiment out of two. Statistical evaluation of the data was performed by using Kruskal–Wallis test. The asterisks above the groups indicate statistical significance in comparison to the vehicle animal and with lines between the respective groups. Brackets above several groups indicate that all included groups were statistically significantly different compared to the corresponding group outside of the bracket (*p ≤ 0.05, **p ≤ 0.01; ***p ≤ 0.001; ****p ≤ 0.0001; LOD, limit of detection at 50 FFU for viral load; *n* = 6).

To test the protective efficacy of the different vaccines and regimen, the mice were challenged with 10^6^ FFU RSV and scored for 5 days (**Figure 1**). Severe clinical symptoms were absent (**Supplementary Figure S5**). At 5 days after challenge, the animals were sacrificed to quantitatively analyze the viral load in lung homogenates. The DD-LEEI-RSV group showed a statistically significant 352-fold reduction of RSV-RNA copy numbers in lungs, only surpassed by the LEEI-RSV i.m. group with a 2.7-fold higher reduction (**Figure 4A**). The FI-RSV group showed a 104-fold and the PC-LEEI-RSV group a 61-fold reduction, both of which were not statistically significant (**Figure 4A**).

#### 3.2.3 Mucosal immunization solely led to IgA antibodies in BALF

The IgG antibodies in the BALF after challenge were found in all immunized groups, as expected, whereby PC-LEEI-RSV (878-fold) and LEEI-RSV i.m. (1,280-fold) induced statistically significantly higher amounts than vehicle-vaccinated animals for binding RSV-A and RSV-B (**Figures 4B, C**). DD-LEEI-RSV had a notable 704-fold (RSV-A) or 901-fold (RSV-B) induction compared to vehicle and FI-RSV i.m. 438-fold (RSV-A) or 697-fold (RSV-B), respectively.

In contrast, RSV-A-binding IgA antibodies were significantly induced by PC-LEEI-RSV (56-fold) and DD-LEEI-RSV (91-fold) compared to the vehicle, whereby DD-LEEI-RSV induced significantly more IgA than the two i.m. groups (**Figure 4B**). For RSV-B-binding IgA antibodies, both i.n. groups induced statistically significantly more antibodies than the vehicle and the FI-RSV i.m. group (**Figure 4C**). These IgA antibody data are consistent with the serum data and highlight the exclusive IgA production after mucosal vaccination.

### 3.3 Immune system after challenge

The exclusion of adverse immunological effects became essential for RSV vaccines after the adverse outcome of a vaccination trial in the 1960s (28). The overwhelming immune response was, among others, associated with a Th2-like immune response in vaccines (28).

#### 3.3.1 IgE antibodies and histopathology induction in i.m. vaccinated animals

**Figure 5:**
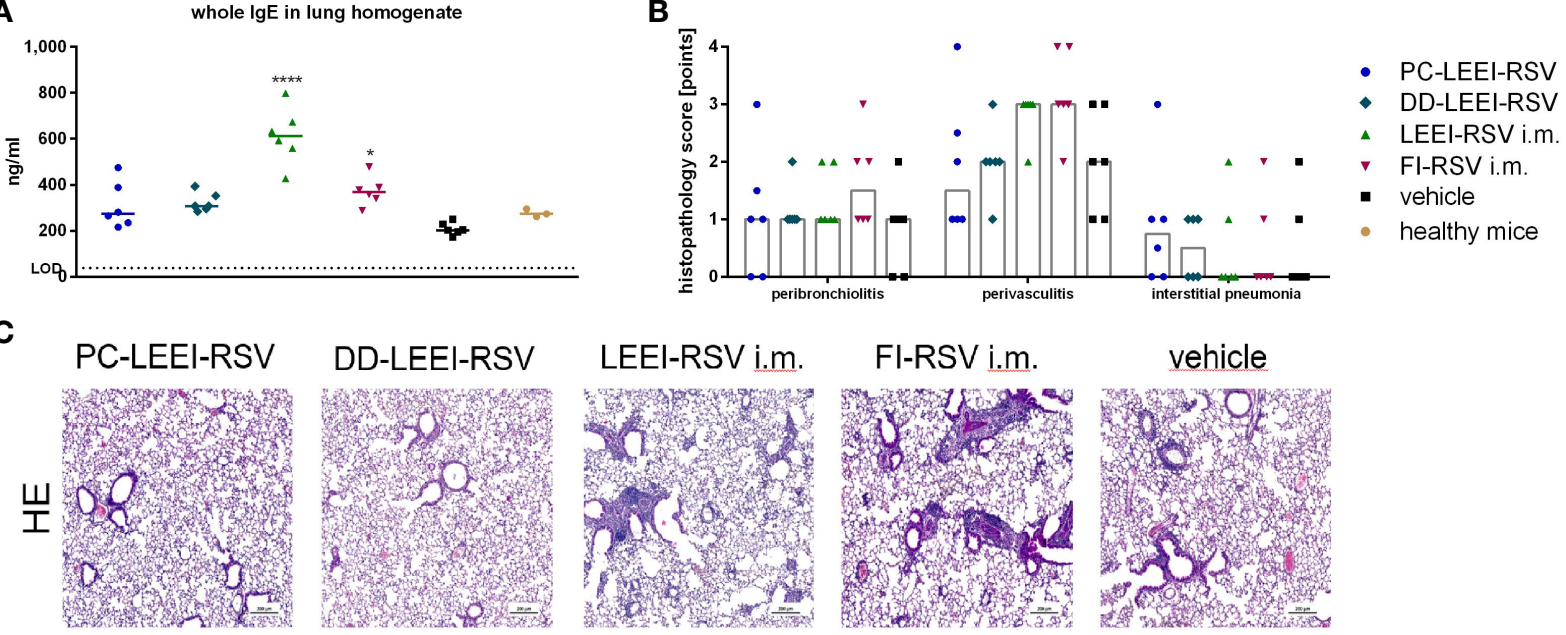
Lung IgE antibodies and H&E staining of lung tissue of immunized and challenged animals. BALB/c mice were vaccinated and challenged as described in **Figure 1**. After euthanasia, the right lung lobes were taken, and IgE-antibodies from lung homogenates were determined by using ELISA **(A)**. The left lung lobes were histologically examined after H&E staining for peribroncholitis, perivasculitis, and interstitial pneumonia (0: non, 1: minimal, 2: mild, 3: moderate, 4: severe; **(B)**). In **(C)**, representative examples for each analyzed group of H&E-stained lung tissues are given (scale = 200 µm). Statistical evaluation of the data was performed by using Kruskal–Wallis test. The asterisks above the groups indicate statistical significance in comparison to the vehicle animal (*p ≤ 0.05, ****p ≤ 0.0001; *n* = 6).

In this regard, lung homogenates were analyzed for IgE levels after RSV challenge. Both i.m. vaccinated groups, LEEI-RSV i.m. (threefold) or FI-RSV i.m. (twofold), induced statistically significantly higher levels of IgE than the vehicle animals (**Figure 4A**). In contrast, the IgE amounts of both mucosally vaccinated groups were only slightly (1.5-fold) elevated compared to the vehicle (**Figure 5A**) and comparable to untreated control BALB/c mice (healthy control, **Figure 5A**).

Further analyses were performed by the pathological assessment of histologically prepared lung tissue after H&E staining. Peribronchiolitis was obviously detectable only in the FI-RSV i.m. group showing elevated levels compared to the other groups (**Figure 5B**). For interstitial pneumonia, the two i.n. groups showed slightly higher scores than the other groups (**Figure 5B**). In addition, the two i.m. groups showed visibly higher levels of perivasculitis than the other groups, and the two i.n. groups seemed to have lower effects than the vehicle animals (**Figure 5B**). This reduced inflammation observed by determining perivasculitis and peribronchiolitis in mucosally vaccinated animals is presented by representative lung sections as shown in **Figure 5C**.

The absence of strong adverse or tissue-harming effects of DD-LEEI-RSV was also observed in human PCLS experiments (**Supplementary Figure S6**).

#### 3.3.2 Th2 cytokine secretion elevated after i.m. vaccination

**Figure 6:**
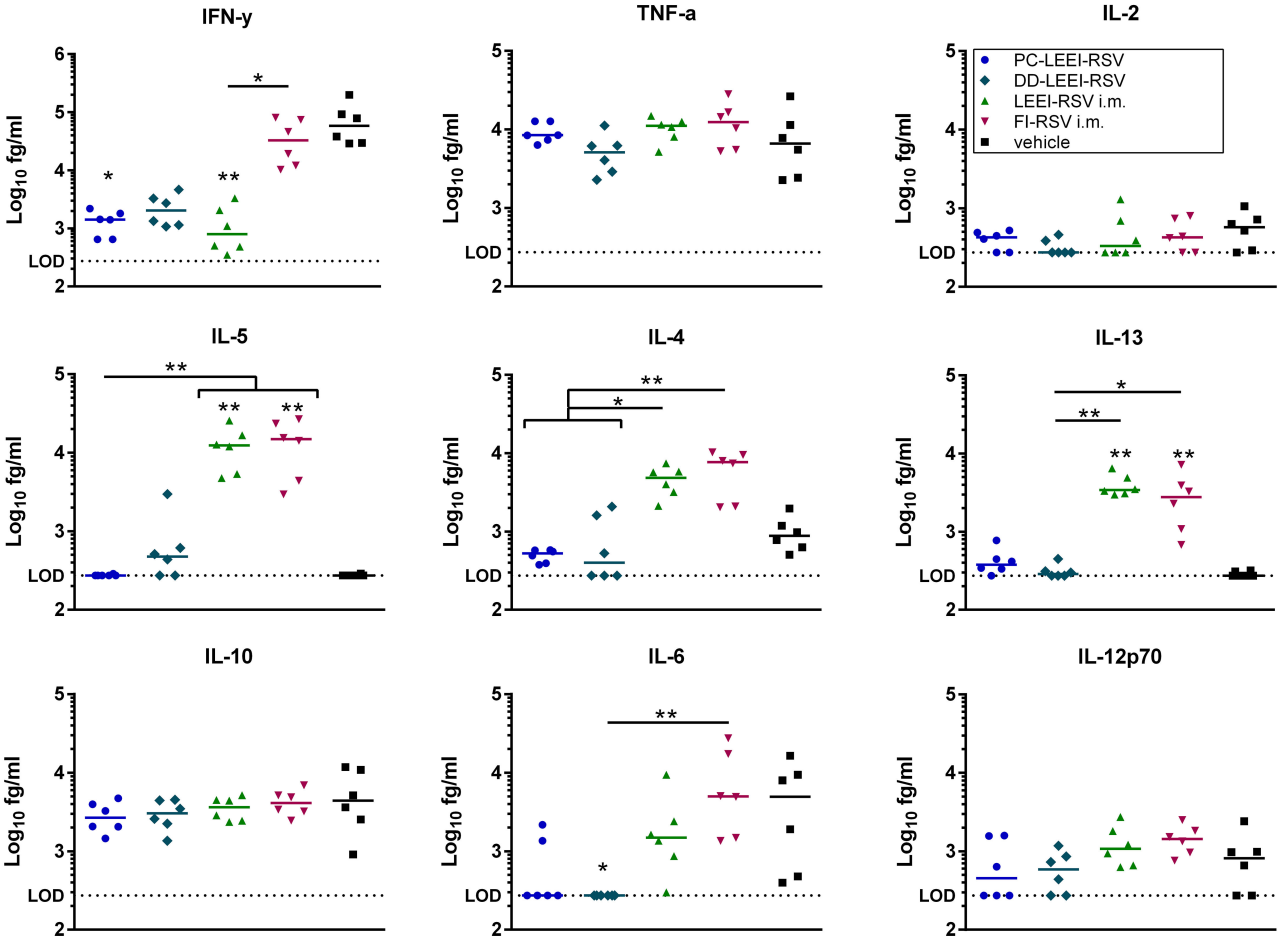
Quantification of cytokines in BALFs of immunized and RSV-infected mice. BALB/c mice were treated as described in **Figure 1**. Bronchoalveolar lavage fluid (BALF) was obtained and analyzed for secreted cytokines. The cytokine levels of IFN-γ, IL-10, IL-6, TNF-α, IL-5, IL-2, IL-4, IL-13, and IL-12p70 were quantified using a bead-based multiplex assay. Limit of detection was defined at 273.42 fg/mL. Data points represent the cytokine level with the median of each group. Statistical evaluation of the data was performed by using Kruskal–Wallis test. The asterisks above the groups indicate statistical significance in comparison to the vehicle group, and the significance between groups is indicated by lines. Brackets above several groups indicate that all included groups are significant compared to the corresponding group outside of the bracket. *p ≤ 0.05, **p ≤ 0.01; *n* = 6.

Additionally, cytokines of BALF were analyzed after vaccination and challenge (**Figure 6**). Interestingly, all three LEEI-RSV-groups showed lower levels of IFN-γ with PC-LEEI-RSV showing 57-fold, DD-LEEI-RSV 33-fold, and LEEI-RSV i.m. 60-fold reduction compared to the vehicle group. The FI-RSV i.m. vaccinated animals had only slightly lower levels of IFN-γ which were statistically significantly higher than LEEI-RSV i.m. animals (31-fold). The DD-LEEI-RSV mice secreted significantly less IL-6 than the vehicle (22-fold) and FI-RSV i.m. group (35-fold). The PC-LEEI-RSV group secreted less IL-6 than the LEEI-RSV i.m. animals (fourfold) and the vehicle group (eightfold). TNF-alpha and IL-12p70 showed only slight changes, and IL-2 and IL-10 had no visible effects.

For the Th2 cytokines IL-4, Il-5, and IL-13, higher levels of cytokines were secreted in the i.m. groups. The LEEI-RSV i.m. showed a statistically significant 10-fold increase in IL-4 compared to the PC-LEEI-RSV group and sixfold to the DD-LEEI-RSV group. The FI-RSV i.m. group exhibited a statistically significant 13-fold increase in IL-4 compared to the PC-LEEI-RSV group and eightfold to the DD-LEEI-RSV group. The mucosally vaccinated mice secreted less IL-4 than the vehicle group with a twofold decrease for PC-LEEI-RSV and a 1.2-fold decrease for DD-LEEI-RSV. Regarding IL-5, the LEEI-RSV i.m. and FI-RSV i.m. vaccinations induced statistically significant increases of 47- and 53-fold, respectively, compared to the vehicle group and PC-LEEI-RSV animals. However, for DD-LEEI-RSV, single animals secreted lower levels of IL-5 than i.m. groups with a 15-fold increase for LEEI-RSV and a 17-fold increase for FI-RSV. For IL-13, significantly higher levels were measured in the i.m. mice compared to the vehicle group (14-fold increase for LEEI-RSV and 11-fold increase for FI-RSV) and the DD-PC-LEEI animals (ninefold increase LEEI-RSV and sevenfold increase FI-RSV) (**Figure 6**).

#### 3.3.3 Balanced immune cell composition with mucosal vaccines

**Figure 7:**
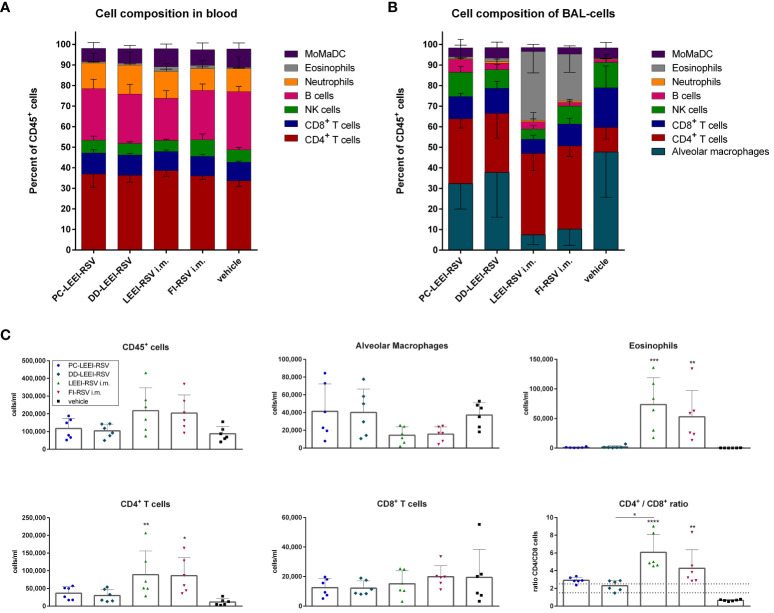
Analysis of cell composition after challenge with flow cytometry. BALB/c mice were vaccinated and challenged as described in **Figure 1**. Cell composition of blood **(A)** and bronchoalveolar lavage (BAL) **(B)** were determined by flow cytometrical analysis. The cell groups were defined as shown in **Supplementary Figure S2** (for **(A)**) and **Supplementary Figure S3** (for **(B)**). MoMaDC indicates clustered cell groups containing monocytes, macrophages, and dendritic cells. In **(C)**, the cell counts per milliliter for the indicated cell groups of the BAL cells is depicted. Shown is the mean ( ± SD) of each group. Statistical evaluation was performed by using Kruskal–Wallis test. The asterisks above the groups indicate statistical significance in comparison to either the respective vehicle group indicated by asterisks directly over the groups or against each other as indicated by lines. (*p ≤ 0.05, **p ≤ 0.01; ***p ≤ 0.001; ****p ≤ 0.0001; *n* = 6).

Moreover, the composition of the immune cells in the blood and BAL was analyzed after vaccination and challenge. In the blood, only eosinophils were statistically significantly higher in LEEI-RSV i.m. (2.4%) and FI-RSV i.m. (1.7%) vaccinated animals compared to the vehicle group (0.6%) upon infection (**Figure 7A**; **Supplementary Table S3**).

The composition of immune cells in the BAL revealed more significant differences (**Figure 7B**). Both i.m. groups had statistically significantly fewer alveolar macrophages (AM) (7.5% LEEI-RSV; 10% FI-RSV) than the vehicle group (48%) (**Supplementary Table S3**) and significantly more CD4^+^ T cells (40% LEEI-RSV, 41% FI-RSV, and 12% vehicle) and eosinophils (34% LEEI-RSV, 23% FI-RSV, and 0.3% vehicle) (**Supplementary Table S3**). Compared to the vehicle group, both i.n. groups had more CD4^+^ T cells (31% PC-LEEI-RSV and 29% DD-LEEI-RSV) (**Figure 6B**). Furthermore, it is worth mentioning that PC-LEEI-RSV (32%) had fewer AM than the DD-LEEI-RSV group (38%) (**Figure 7B**). LEEI-RSV i.m. had significantly less CD8^+^ T cells (7%) and NK cells (5%) than the vehicle (19% CD8^+^ T cells and 12% NK cells) and significantly fewer NK cells and more eosinophils than PC-LEEI-RSV mice (12% and 0.9%, respectively) (**Supplementary Table S3**).

Regarding immune cell counts per milliliter, the two i.m. groups had more lymphocytes (CD45^+^ cells) than the other groups and fewer AMs, with DD-LEEI-RSV animals having the lowest lymphocyte count (1 × 10^5^ cells/mL) among the vaccinated animals, indicating the lowest immune cell infiltration (**Figure 6C**). The eosinophil counts (324-fold LEEI-RSV and 233-fold FI-RSV) and CD4^+^ T cell counts (7.7-fold LEEI-RSV and 7.5-fold FI-RSV) in i.m. vaccinated mice were statistically significantly higher than the vehicle group and visibly higher than the i.n. groups (29- to 71-fold). The differences of CD8^+^ T cell numbers were not significant between all groups (**Figure 7C**).

The ratio of CD4^+^ to CD8^+^ T cells was calculated, as imbalanced ratios have been associated with adverse effects (34, 35). A ratio of 1.5 to 2.5 is considered balanced, with higher values indicating an over-bursting and lower values indicating an impaired immune system (35). The i.n. vaccine candidates had ratios within the balanced range, indicating a balanced but activated immune system (**Figure 7C**). In comparison, the mean ratios of the i.m. groups were 6.1 (LEEI-RSV) and 4.3 (FI-RSV), exceeding the balanced immune response (**Figure 7C**). In contrast, the mean CD4^+^/CD8^+^ ratio of the vehicle group was 0.7, which could be explained by the experiment duration of 5 days in which a CD4^+^ T cell induction is not possible. In summary, the calculations of the CD4^+^/CD8^+^ T cell ratios underline the imbalanced immune responses detected in animals due to the i.m. vaccination with either Alhydrogel-adjuvanted LEEI-RSV or FI-RSV and more balance after i.n. vaccination.

## 4 Discussion

Mucosal vaccines are advantageous as they induce mucosal immunity and secretory IgA, which are essential for the defense of respiratory pathogens (15, 36–38). Clinical data suggest that the local immune system plays an important role in controlling RSV infections (11). Recently, vaccines against RSV have been approved for adults (5, 6), but the question remains as to whether a mucosal vaccine, which protects at the site of infection, could provide additional benefits. Combining mucosal with approved vaccines could induce long-term protection (36, 39, 40).

Regarding an i.n. vaccine against RSV, we have developed an optimized, formulated inactivated RSV vaccine that aims to protect mice and prevent imbalanced immune responses. DD-LEEI-RSV was able to protect mice with relatively low levels of systemic binding and neutralizing antibodies but induced balanced immune responses reflected in a non-pathological BAL cell composition and cytokine levels. The absence of IFN-γ could be due to reduced viral replication during the initial infection phase, resulting in lower cytokine induction. Similar immunological effects were observed with PC-LEEI-RSV. However, the reduction in RSV copies was 5.8 times lower than with DD-LEEI-RSV and not significant compared to the vehicle control. One possible explanation for this difference could be the molecular properties of the liposomes since PC is anionic whereas DD is cationic. These charge differences could influence the uptake of liposomes by AMs, leading to more efficient antigen presentation with positively charged DD compared to negatively charged PC (41).

For FI-RSV, a clinical trial in the 1960s showed enhanced disease symptoms and two fatal cases in FI-RSV-vaccinated toddlers after natural infection (28). These adverse effects are thought to be due to the misfolding of the RSV-F protein, resulting in a poor and imbalanced immune response, absence of RSV-neutralizing antibodies, antibody-dependent enhancement, and a Th2 bias (42, 43). We observed adverse immunological effects in mice immunized with i.m. applied FI-RSV and LEEI-RSV after challenge. These effects included high IgE levels, severe lung damage (**Figure 5**), and high levels of Th2-related cytokines (**Figure 6**) despite nearly undetectable viral replication and robust humoral immune responses (**Figures 3**, **4**). In the Th2 response, Th2 cells produce IL-4, which leads to a class switch to IgE-producing B cells and further Th2 cell priming, and IL-5, leading to eosinophil activation and recruitment. These Th2-biased immune responses have been linked to lung pathologies (44) which we also observed (**Figure 5**). We showed earlier that the choice of adjuvant is a critical factor regarding Th2 bias as the i.m. vaccination with LEEI-inactivated RSV in combination with Alhydrogel induces a Th2 immune response in mice (20). Despite a reduced Th2 bias when using the same vaccine and vaccination route but other adjuvants (20), we decided to use Alhdyrogel to enable a comparison with FI-RSV. It is known that Alhydrogel induces a Th2 bias (20, 44) and suboptimal T cell responses (36) which could contribute to the adverse immunological effects observed. We hypothesize that the use of Alhydrogel and the i.m. vaccination route induced a Th2 immune response associated with enhanced inflammation after RSV challenge. Importantly, both formulated mucosal LEEI-RSV vaccines did not show Th2-biased or misbalanced immune responses. It is worth mentioning that the vaccination routes cannot be compared directly head to head as the packaging, application route, and application frequency differ. However, the LEEI-RSV i.m. vaccination group was included as a positive control for protective efficacy, which was shown before (19). However, the protective efficacy using mucosal vaccination independent on the application frequency is promising and might open a new option for the effective protection against RSV.

In the presented vaccination trial, only the mucosal vaccines induced RSV-specific IgA antibodies. IgA antibodies are known to be present mainly on the mucosa. Previous studies have shown that RSV-specific IgA antibodies are important correlates of protection and can help to reduce the severity of infection (8, 9, 37, 38), highlighting the significance of the findings in the present study. Consequently, the antibodies in the BALF may provide a first-line defense by binding and neutralizing RSV, whereas serum-neutralizing antibodies first need to transudate through the lung mucosa (36).

To induce protection against mucosal pathogens directly at the site of infection, mucosal vaccines have become increasingly popular. In this approach, it is important to consider strategies to overcome the mucus barrier such as multiple applications, the use of optimized mucosal formulations like liposomes or lipid nanoparticles, or the use of infection- and replication-competent vaccines. In this regard, LAAVs or other virus vectors have shown promising results in several preclinical and clinical trials (8, 9, 12–15, 40, 45, 46). LAAVs mimic natural virus infection and thereby activate both the innate and the adaptive immune systems (13, 15, 45, 46). Currently, only i.n. LAAV vaccines against influenza are approved, which are well tolerated and, in terms of efficacy, with cross-protective effects against drifted influenza strains (15). For RSV, some LAAV vaccines are in phase 1 clinical trials but require further extensive testing to exclude rare events as a case of lower respiratory illness was observed (13). The history of RSV-LAAV is characterized by either severe disease symptoms due to insufficient attenuation (46) or insufficient protection due to over-attenuation (45).

Another replication-competent vaccine technology for mucosal application is the use of different viral vectors (8, 9, 12, 14, 39, 40). Adenoviral vectors (AdV), already approved for i.m. vaccination against SARS-CoV 2 (47), have been shown to induce superior protective efficacy when applied mucosally compared to i.m. application (9, 12, 40). In the context of RSV, an AdV vaccine induced higher protection than a natural infection in mice, with better cellular and humoral immune responses (8). Other vectors, such as the modified vaccinia Ankara virus (MVA) vector, have also been used in mucosal applications, showing strong antibody induction and protection against RSV in mice (9, 14).

Despite these promising approaches, there are several disadvantages associated with replication-competent vaccines. Pre-existing immunity against the vaccines may reduce the efficacy, and cases of reversion to a virulent pathogen or retrograde transmission to the brain have been reported (16–18, 45–47). In addition, AdV vaccines against SARS-CoV-2 have rarely been associated with a severe side effect of immune thrombotic thrombocytopenia, leading to concerns regarding their safety (47).

Non-replicating, inactivated vaccines, on the other hand, do not pose these safety concerns, making them potent candidates for mucosal application against respiratory viruses (11, 19, 48–50). This has been demonstrated for an irradiated H1N1 influenza vaccine which showed protective efficacy only after mucosal application and not through systemic routes (48). In this i.n. vaccination study, cross-reactive CD8^+^ T cells were induced, and a heterologous challenge with lethal H5N1 was cleared (48). Moreover, a chemically inactivated SARS-CoV-2 vaccine approach has shown high levels of IgA after i.n. vaccination, providing better protection in the URT compared to systemic immunization and potentially enabling cross-protection (49). Even though we saw a viral load reduction after i.m. vaccination, cross-reactive RSV-specific IgA antibodies were solely detected in i.n. vaccinated animals which correlated with protection against severe lung pathology. Furthermore, we detected a healthier distribution of immune cells in the BAL with 38% AM of CD45^+^ cells in DD-LEEI-RSV-vaccinated mice and a balanced ratio of 2.3 between CD4^+^ and CD8^+^ T cells. It is known that mucosal vaccines can induce lung-specific resident memory CD4^+^ and CD8^+^ T cells, and the presence of lung-specific resident memory CD4^+^ T cells is particularly important for long-term immunity (36).

Our lipid formulation using DD and RSV-LEEI demonstrated a protective efficacy in mucosally vaccinated mice and induced a balanced immune response visible in cell composition and cytokine secretion. Additional adjuvanting could further enhance the protective efficacy. Promising options include pattern recognition receptor ligands such as cyclic di-nucleotides (51) or LP-GMP (36), detoxified enterotoxins such as CTA1-DD (11), or cytokines such as IL-1β or IL-12 (8, 11). These adjuvants have shown improved protective efficacy in mucosal applications against RSV (8), influenza (11, 51) or SARS-CoV-2 (36). However, it is important to note that the usage of additional adjuvants may also result in unwanted side effects such as facial paralysis or diarrhea (11).

A limitation of the presented study is that T cells were not further distinguished into resident, circulating, or memory T cells to assess the durability of protection (36). Additionally, evaluating the effects of the vaccine without challenge could give interesting insights but was not the focus of this study, as the main objective was the comparison with FI-RSV and the immune response after challenge.

DD-LEEI-RSV is a promising i.n. vaccine candidate for protection against RSV. As i.n. vaccination in humans can be self-administered with minimal medical assistance, this approach might have implications for various respiratory viruses with temporary immunity and might be an option for booster vaccinations. We have developed a potent vaccine that has shown superior protection compared to our previous approaches and provided new insights into the immunological impact of our vaccine after a virus challenge. In summary, our study suggests that LEEI-RSV formulated with DD may be a valuable approach for protecting humans against RSV.

## Data availability statement

The original contributions presented in the study are included in the article/**Supplementary Material**. Further inquiries can be directed to the corresponding author.

## Ethics statement

The studies involving human tissues were approved by the Ethical Committee of the Hannover Medical School. The studies were conducted in accordance with the local legislation and institutional requirements. The human samples used in this study were acquired from lung tissue from cancer patients with written informed consent for the use of such tissue for research. Written informed consent for participation was not required from the participants or the participants’ legal guardians/next of kin in accordance with the national legislation and institutional requirements. The animal study was approved by Landesdirektion Sachsen, Germany, and the Ethical Committee for Animal Experiments of Leipzig, Germany. The study was conducted in accordance with the local legislation and institutional requirements.

## Author contributions

VE: Conceptualization, Data curation, Formal analysis, Investigation, Methodology, Visualization, Writing – original draft, Writing – review & editing. SR: Data curation, Formal analysis, Investigation, Writing – review & editing. JFi: Formal analysis, Investigation, Writing – review & editing. JB: Methodology, Writing – review & editing. YM: Methodology, Writing – review & editing. JK: Methodology, Writing – review & editing. FL: Validation, Writing – review & editing. JD: Project administration, Supervision, Validation, Writing – review & editing. SS: Project administration, Supervision, Validation, Writing – review & editing. CH: Methodology, Project administration, Supervision, Validation, Writing – review & editing. MT: Methodology, Project administration, Supervision, Validation, Writing – review & editing. JFe: Project administration, Supervision, Validation, Writing – review & editing. SU: Project administration, Validation, Writing – review & editing. TG: Conceptualization, Funding acquisition, Project administration, Supervision, Validation, Writing – original draft, Writing – review & editing.
