# Supplementary material for: Mucosal immunization with a low-energy electron inactivated respiratory syncytial virus vaccine protects mice without Th2 immune bias
Source: Front Immunol. 2024 Apr 5;15:1382318. doi: 10.3389/fimmu.2024.1382318 (PMC11026718; doi:10.3389/fimmu.2024.1382318)
Supplement: Supplementary file 1 [file DataSheet_1.docx]

Supplementary Material

# Supplementary Figures


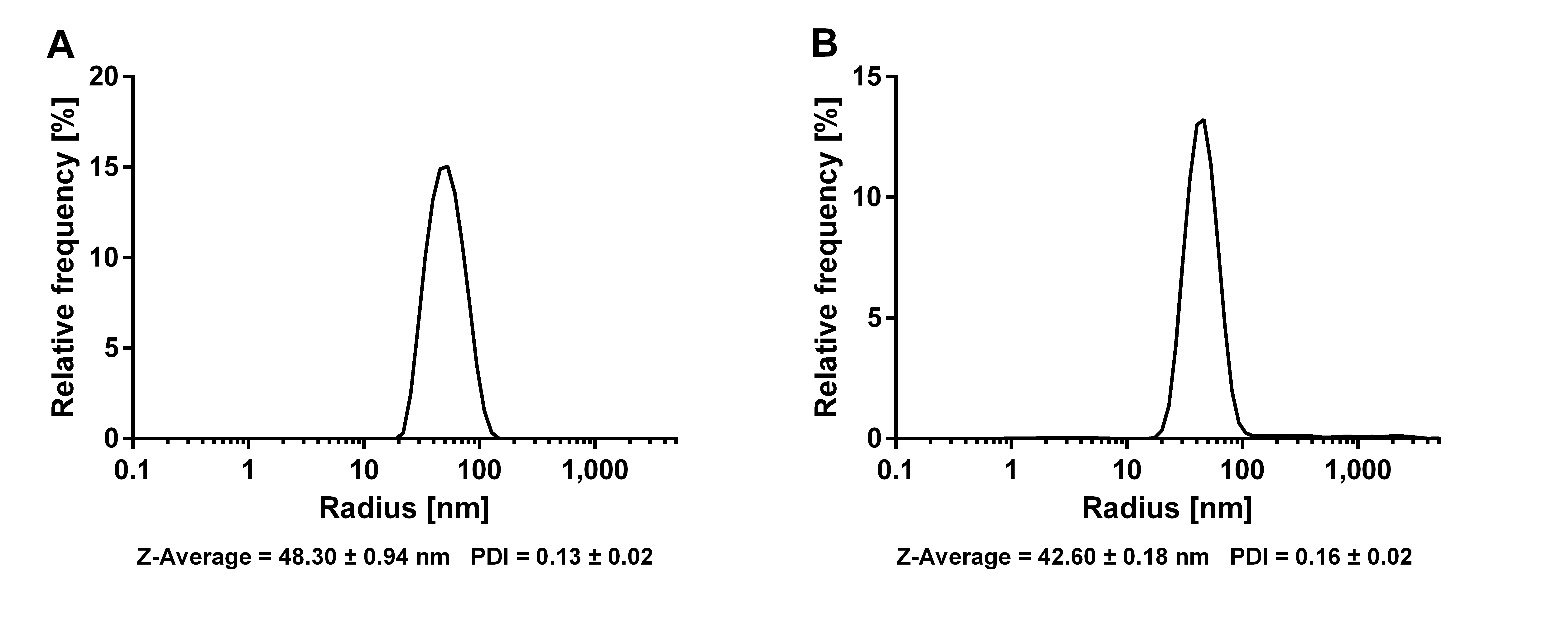


**Supplement-Figure 1 –** **Size analysis of DD-liposomes.** Size distribution, Z-average and PDI (polydispersity index) were measured by Zetasizer (A) and Prometheus Panta (B). Small differences between the devices were observed and might occur due to different scattering angles or not perfectly round particles.

**
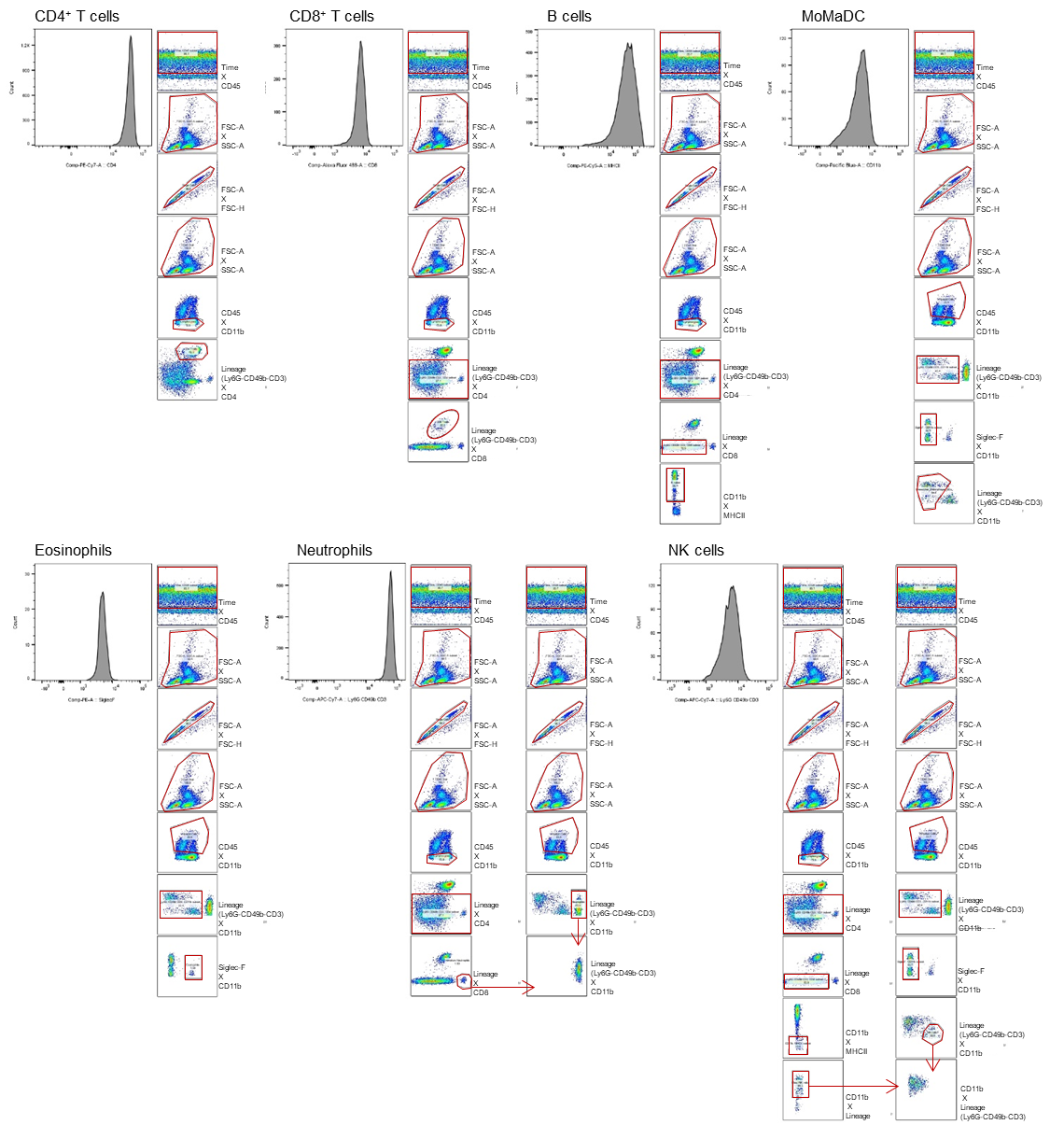
Supplement-Figure 2 – Gating strategy for the analyses of PBMCs (peripheral blood mononuclear cells) from blood.** BALB/c mice were treated as described in Supplement Fig. 2. At the time point of euthanasia blood was collected for cell composition analysis by flow cytometry and an exemplary gating strategy is presented. For all cell types first the lymphocytes were defined, by choosing CD45-positive cells in the time gate to exclude inadequate measurement quality and CD45-negative events. From this a single cell exclusion was done (FFS-A vs. FFC-H) and lymphocytes were defined according to their size distribution (FSC-A vs. SSC-A). From the lymphocyte population indicated cell types were distinguished. Namely CD4 + T cells by gating on CD11b-negative cells and then CD4- and CD3-postitve cells (CD45+, CD11b-, CD4+, lineage+ (CD3)). From the non CD4 + T cells, CD8 + T cells were defined as CD8- and CD3-positive cells (CD45+, CD11b-, CD4-, CD8+, lineage+ (CD3)). From the non-CD8 + T cells and linage-negative/dim cells B cells were defined as MHCII-positive cells (CD45+, CD11b-, CD4-, CD8-, lineage-/dim, MHCII+). Neutrophils were defined as a combination of immature neutrophils that were from the CD8-negative population the linage-high population (CD11b-, CD4-, CD8-, lineage+++ (Ly6G)) and the mature neutrophils that are from the lymphocyte population the CD11b-positive cells, that are lineage-high (CD45+, CD11b+, lineage++). From non-neutrophils eosinophils were defined as Siglec-F-positive (CD45+, CD11b+, lineage-Siglec-F+). From the non-eosinophils the cell group of monocytes, macrophages and dendritic cells (MoMaDC) was defines as lineage–negative/low cells (CD45+, CD11b+, Siglec-F-, lineage-/low). The NK cells were defined as a combination of the CD11b-neagtiv non-B cells that are lineage-positive and the non-MoMaDC that are lineage positive (CD45+, CD4-, CD8-, MHCII-, Siglec-F-, lineage+ (CD49b)).


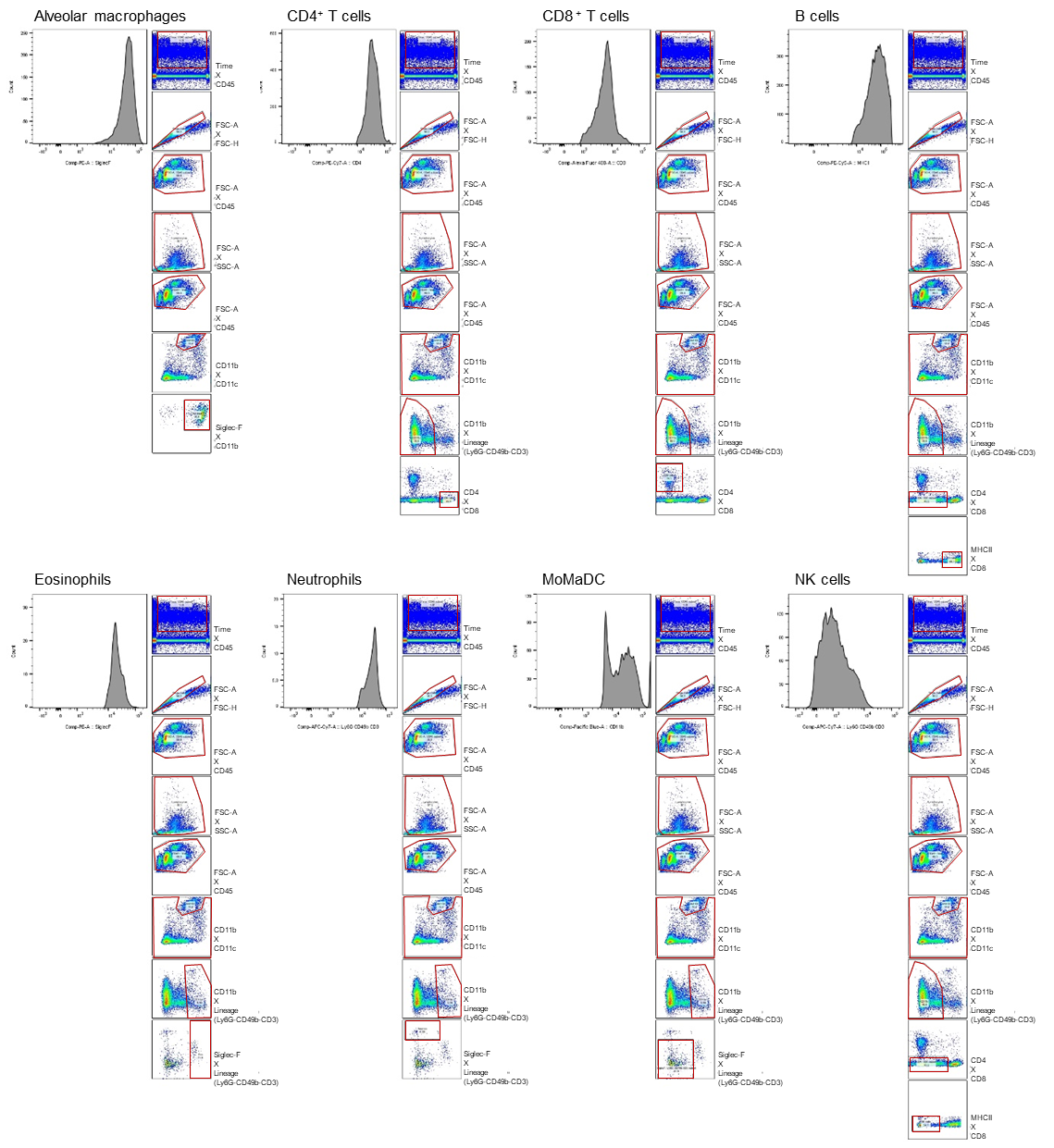


**Supplement-Figure 3 – Gating strategy BAL cells.** BALB/c were treated as described in Supplement Fig. 2 and at euthanasia bronchoalveolar lavage (BAL) was collected for flow cytometrical analysis of cell composition. To identity different cell types an exemplary gating strategy is presented. For all cell types first the lymphocytes were defined, by choosing CD45-positive cells in the time gate to exclude inadequate measurement quality and CD45-negative events. From this a single cell exclusion was done (FFS-A vs. FFC-H) and a pre-lymphocyte-gate was defined (FSC-A, CD45+). From this, cells were gated according to the size distribution (FSC-A vs. SSC-A). For a final lymphocyte gate again cell size was analyzed against CD45 and CD45-positive cells were chosen. As alveolar macrophages (AM) have a high autofluorescence they were defined and excluded before any other cell type was defined. Therefore, AM were defined as CD11b-positive and CD11c-high cells and for a final definition also only Siglec-F-positive were chosen as AM (CD45+, CD11b+, CD11c++, Siglec-F+). From the non-AM cells CD4 ^+^ T cells were defined as CD11b-negative/dim, CD4-positive and CD8-negative cells (CD45+, CD11b-/dim, CD8-, CD4+) and CD8 ^+^ T cells as CD11b-negative/dim, CD8-positive and CD4-negative cells (CD45+, CD11b-/dim, CD4-, CD8+). From the non-CD4 ^+^ or –CD8 ^+^ T cells on the one side B cells were defined as MHCII-positive cells (CD45+, CD11b-/dim, CD8-, CD4-, MHCII+) and on the other NK cells as the MHCII- population (CD45+, CD11b-/dim,
CD8-, CD4-, MHCII-, lineage+). For the myeloid cell lineage from the non-AM the CD11b-positive population was used to define neutrophils as lineage-highly positive (CD45+, CD11b+, lineage++ (Ly6G), Siglec-F-) and eosinophils as SiglecF-positive cells (CD45+, CD11b+, Siglec-F+). The non-neutrophils or –eosinophils are defined as the group of monocytes, macrophages and dendritic cells (MoMaDC) (CD45+, CD11b+, lineage-/dim, Siglec-F-) and not further separated.


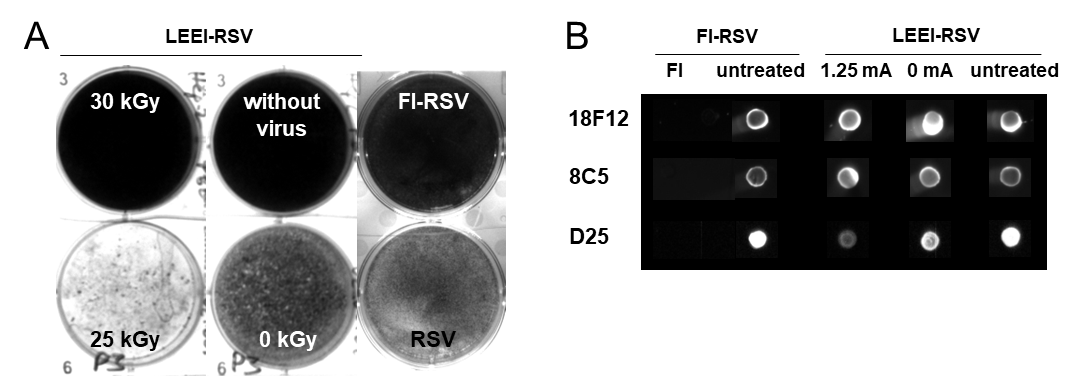


**Supplement-Figure 4 – Antigen conservation and infectivity test of irradiated and formalin treated RSV.** The conservation of RSV-F (18F12), prefusion RSV-F (D25) and RSV-G (8C5) of different RSV samples, inactivated as indicated, were measured with Dot Blot analysis (A). Detection time periods were optimized and for 18F12 10 minutes, for 8C5 5 minutes and for D25 40 minutes, respectively. RSV samples were incubated on HEp2-cells after LEEI or FI treatment to test the residual infectivity. Six days after cultivation the resulted supernatants were used for infection of fresh HEp-2 cells and incubated for six days. This passaging was performed two more times and stained with crystal violet to visualize the cytopathic effect (CPE). As negative control cells were mock infected with virus-free medium (without virus) or as positive control with untreated RSV (RSV). Shown are the plates after third passage (B). Solid dark wells represent completely stained cells with no detectable CPE, whereby in clear wells the cell layer was destroyed by the viral infection.


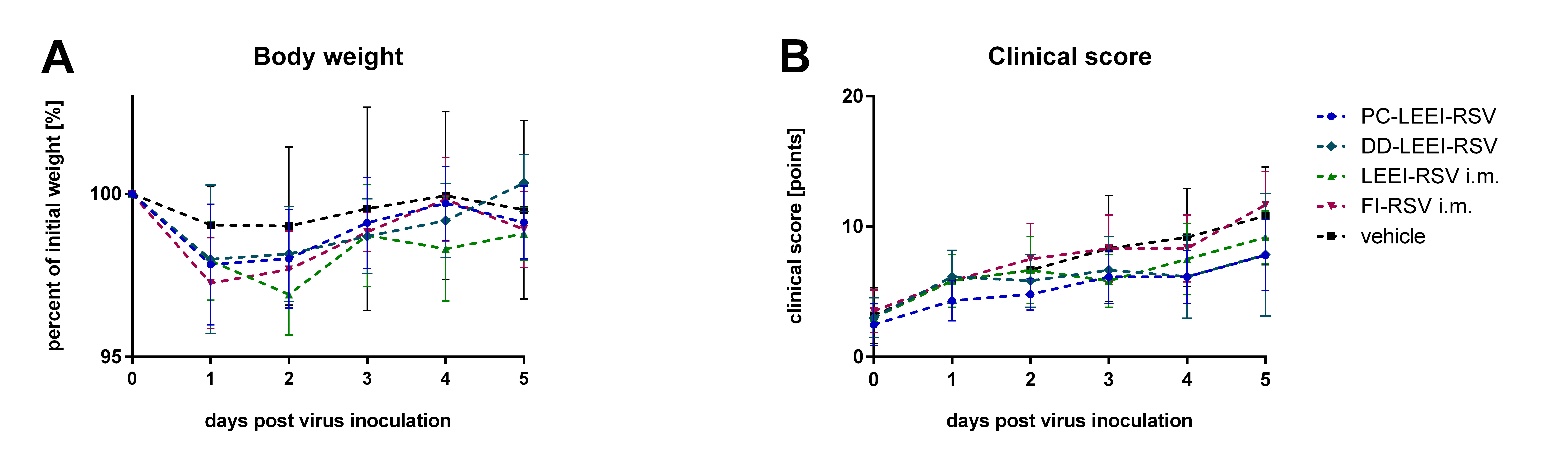


**Supplement-Figure 5 – Clinical score and weight loss after RSV infection.** BALB/c mice were treated as described in Supplement Figure 2. Shown are the clinical score (A) and the weight loss (B) of the different vaccination groups. (n=6).


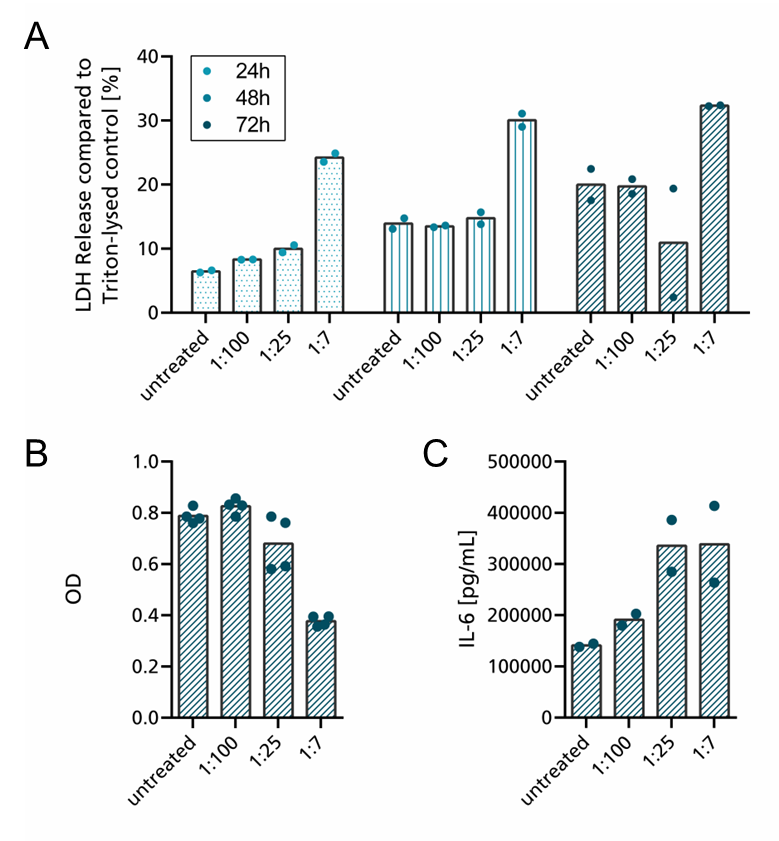


**Supplement-Figure 6 – Analysis of adverse effects of the DOTAP/DOPE-vaccine in hPCLS.** Human PCLS were incubated for 72 hours with different concentrations of DD-LEEI-RSV or left untreated. At indicated timepoints samples were analyzed for tissue viability and cytokine release. Release of lactate dehydrogenase measured in culture medium at 24h, 48 and 72h (A). Measured values were normalized to Triton lysed control (1% Triton X-100). Metabolic activity of the tissue measured by WST-1 assay after 72h tissue culture (B). Secretion of IL-6 into culture supernatants after DD-LEEI-RSV treatment or untreated tissue measured by ELISA after 72h tissue culture (C). Single dots represent measures of individual wells and bars indicate indicates the mean of replicate measures. (n=1).





**Supplement-Figure 7 – Cell composition per mL after challenge.** BALB/c mice were treated as described in Supplement Fig. 2. Cell composition per mL for indicated cell groups of the BAL-cells is depicted. MoMaDC were clustered cell groups containing monocytes, macrophages and dendritic cells. Shown is the mean (+/- SD) of each group. (n=6).

# Supplementary Tables

Supplement-Table 1: Statistical analysis of the RSV specific humoral immune response between the analyzed groups (p-values are listed)

|  | groups ^(1)^ | | serum IgG | | | serum IgA | | | VNT | | |
| --- | --- | --- | --- | --- | --- | --- | --- | --- | --- | --- | --- |
|  |  |  | pre | prime | boost | pre | prime | boost | pre | prime | boost |
| RSV-A | vehicle | PC | >0.9999 | 0.0022 | 0.0022 | 0.5152 | 0.0152 | 0.0022 | >0.9999 | >0.9999 | 0.0022 |
|  |  | DD | 0.9740 | 0.0022 | 0.0022 | 0.5087 | 0.0628 | 0.0022 | >0.9999 | >0.9999 | 0.0281 |
|  |  | LEEI | 0.4113 | 0.0022 | 0.0022 | 0.2229 | 0.7944 | 0.3810 | >0.9999 | 0.0606 | 0.0022 |
|  |  | FI | 0.1190 | 0.0022 | 0.0022 | 0.4134 | 0.9156 | 0.0931 | >0.9999 | >0.9999 | 0.0022 |
|  | PC | DD | 0.9805 | 0.0087 | 0.0649 | >0.9999 | 0.0628 | 0.5887 | >0.9999 | >0.9999 | 0.0476 |
|  |  | LEEI | 0.5152 | 0.0260 | 0.0043 | 0.8485 | 0.0628 | 0.0022 | >0.9999 | 0.0606 | 0.0022 |
|  |  | FI | 0.3939 | 0.5887 | 0.5563 | 0.2100 | 0.0606 | 0.0022 | >0.9999 | >0.9999 | 0.0736 |
|  | DD | LEEI | 0.7316 | 0.0022 | 0.0022 | 0.7857 | 0.5455 | 0.0022 | >0.9999 | 0.0606 | 0.0022 |
|  |  | FI | 0.4848 | 0.0043 | 0.0022 | 0.2879 | 0.6017 | 0.0022 | >0.9999 | >0.9999 | 0.0022 |
|  | LEEI | FI | 0.5152 | 0.0087 | 0.0022 | 0.2597 | 0.6991 | 0.6991 | >0.9999 | 0.0606 | 0.0022 |
| RSV-B | vehicle | PC | 0.6991 | 0.0022 | 0.0022 | 0.1017 | >0.9999 | 0.0022 | - | - | - |
|  |  | DD | 0.5584 | 0.0022 | 0.0022 | 0.0325 | 0.3095 | 0.0022 | - | - | - |
|  |  | LEEI | 0.6991 | 0.0022 | 0.0022 | 0.0087 | 0.6212 | 0.0152 | - | - | - |
|  |  | FI | 0.0649 | 0.0022 | 0.0022 | 0.2511 | 0.4848 | 0.0584 | - | - | - |
|  | PC | DD | 0.3680 | 0.0411 | 0.2403 | 0.9740 | 0.2576 | >0.9999 | - | - | - |
|  |  | LEEI | 0.9372 | 0.0087 | 0.0152 | 0.1320 | 0.5887 | 0.0022 | - | - | - |
|  |  | FI | 0.0931 | 0.6991 | 0.6991 | 0.9372 | 0.3312 | 0.0022 | - | - | - |
|  | DD | LEEI | 0.3723 | 0.0022 | 0.0022 | 0.1797 | 0.1320 | 0.0043 | - | - | - |
|  |  | FI | 0.0216 | 0.0260 | 0.0043 | 0.2359 | 0.1429 | 0.0022 | - | - | - |
|  | LEEI | FI | 0.1320 | 0.0087 | 0.0022 | 0.0390 | 0.4848 | 0.4567 | - | - | - |

^(1)^ Statistical analysis are indicated for the group in the second row and the corresponding group of the respective line.

Supplement-Table 2: Statistical analysis of humoral immune response between the different time points of vaccination in each group (p-values are listed)

|  |  | groups ^(1)^ | | PC | DOTAP | LEEI | FI | vehicle |
| --- | --- | --- | --- | --- | --- | --- | --- | --- |
| RSV-A | Serum IgG | prä | prime | 0.0022 | 0.0022 | 0.0022 | 0.0022 | 0.0022 |
|  |  |  | boost | 0.0022 | 0.0022 | 0.0022 | 0.0022 | 0.0022 |
|  |  | prime | boost | 0.0022 | 0.0022 | 0.0022 | 0.0022 | 0.0022 |
|  | Serum IgA | prä | prime | 0.0498 | 0.5498 | 0.3247 | 0.5584 | 0.1234 |
|  |  |  | boost | 0.0022 | 0.0022 | 0.9069 | 0.0866 | 0.6169 |
|  |  | prime | boost | 0.0022 | 0.0022 | 0.3983 | 0.2229 | 0.7879 |
|  | VNT | prä | prime | >0.9999 | >0.9999 | 0.0606 | >0.9999 | >0.9999 |
|  |  |  | boost | 0.0022 | 0.0152 | 0.0022 | 0.0022 | >0.9999 |
|  |  | prime | boost | 0.0022 | 0.0152 | 0.0022 | 0.0022 | >0.9999 |
| RSV-B | Serum IgG | prä | prime | 0.0022 | 0.0022 | 0.0022 | 0.0022 | 0.3939 |
|  |  |  | boost | 0.0022 | 0.0022 | 0.0022 | 0.0022 | 0.1320 |
|  |  | prime | boost | 0.0022 | 0.0022 | 0.0022 | 0.0022 | 0.0411 |
|  | Serum IgA | prä | prime | 0.3939 | 0.5130 | 0.1320 | 0.6991 | 0.0909 |
|  |  |  | boost | 0.0022 | 0.0022 | 0.6667 | 0.1277 | 0.2900 |
|  |  | prime | boost | 0.0022 | 0.0087 | 0.0844 | 0.0649 | 0.4545 |

^(1)^ Statistical analysis are indicated for the group in the second row and the corresponding group of the respective line.

Supplement-Table 3: Statistical analysis of the cell composition in percentages of population of the blood or BAL cells between the vaccination groups after challenge (p-values are listed)

|  | groups ^(1)^ | | AM | CD4+ cells | CD8+ cells | NK-cells | B-cells | Neutrophils | Eosinophils | MoMaDC |
| --- | --- | --- | --- | --- | --- | --- | --- | --- | --- | --- |
| blood  cells | vehicle | PC | - | 0.5298 | 0.1663 | >0.9999 | >0.9999 | >0.9999 | >0.9999 | 0.6871 |
|  |  | DD | - | >0.9999 | >0.9999 | >0.9999 | 0.8810 | >0.9999 | >0.9999 | >0.9999 |
|  |  | LEEI | - | 0.1741 | >0.9999 | >0.9999 | 0.0173 | >0.9999 | 0.0241 | >0.9999 |
|  |  | FI | - | >0.9999 | >0.9999 | >0.9999 | 0.8215 | >0.9999 | 0.1902 | >0.9999 |
|  | PC | DD | - | >0.9999 | >0.9999 | >0.9999 | >0.9999 | >0.9999 | >0.9999 | >0.9999 |
|  |  | L LEEI | - | >0.9999 | 0.5707 | >0.9999 | 0.9438 | >0.9999 | 0.0284 | 0.6155 |
|  |  | FI | - | >0.9999 | >0.9999 | >0.9999 | >0.9999 | >0.9999 | 0.2168 | >0.9999 |
|  | DD | L LEEI | - | >0.9999 | >0.9999 | >0.9999 | >0.9999 | >0.9999 | 0.3880 | >0.9999 |
|  |  | FI | - | >0.9999 | >0.9999 | 0.6631 | >0.9999 | >0.9999 | >0.9999 | >0.9999 |
|  | LEEI | FI | - | >0.9999 | >0.9999 | 0.2171 | >0.9999 | >0.9999 | >0.9999 | >0.9999 |
| BAL  cells | vehicle | PC | >0.9999 | 0.4547 | >0.9999 | >0.9999 | 0.0788 | >0.9999 | >0.9999 | >0.9999 |
|  |  | DD | >0.9999 | 0.4913 | >0.9999 | >0.9999 | 0.1818 | >0.9999 | 0.7931 | >0.9999 |
|  |  | LEEI | 0.0073 | 0.0058 | 0.0131 | 0.0035 | >0.9999 | >0.9999 | 0.0001 | 0.0829 |
|  |  | FI | 0.0285 | 0.0019 | >0.9999 | 0.7661 | >0.9999 | >0.9999 | 0.0020 | >0.9999 |
|  | PC | DD | >0.9999 | >0.9999 | >0.9999 | >0.9999 | >0.9999 | >0.9999 | >0.9999 | >0.9999 |
|  |  | LEEI | 0.0791 | >0.9999 | 0.4545 | 0.0051 | >0.9999 | >0.9999 | 0.0455 | 0.2800 |
|  |  | FI | 0.2366 | 0.8222 | >0.9999 | 0.9445 | >0.9999 | >0.9999 | 0.2918 | >0.9999 |
|  | DD | LEEI | 0.0588 | >0.9999 | 0.0958 | 0.4913 | >0.9999 | >0.9999 | 0.0869 | 0.0958 |
|  |  | FI | 0.1823 | 0.7661 | >0.9999 | >0.9999 | >0.9999 | >0.9999 | 0.4908 | >0.9999 |
|  | LEEI | FI | >0.9999 | >0.9999 | 0.6158 | 0.7131 | >0.9999 | >0.9999 | >0.9999 | >0.9999 |

^(1)^ Statistical analysis are indicated for the group in the second row and the corresponding group of the respective line.
